# Sublingual emphysema following alveoloplasty: A case report

**DOI:** 10.1002/ccr3.3106

**Published:** 2020-07-15

**Authors:** Austin Dodge, Katelyn Kreh, Vrisiis Kofina, Swati Y. Rawal

**Affiliations:** ^1^ Marquette University School of Dentistry Milwaukee WI USA

**Keywords:** alveoloplasty, emphysema, intraoperative complications, mouth floor

## Abstract

Various cases of introduction of air into soft tissues have been reported in the dental literature. Here, we report a rare case of localized sublingual emphysema after alveoloplasty. There was no facial involvement. The patient responded to treatment and recovered uneventfully.

## Graphical abstract

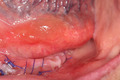

## 1 INTRODUCTION

Subcutaneous and/or submucous emphysema after dental treatment occurs infrequently.^1^ High‐pressure air instruments, such as high‐speed hand pieces, air turbine hand pieces, and air syringes, are mainly responsible for this phenomenon.^2^, ^3^ The condition is characterized by air being forced underneath the tissue leading to swelling, crepitus on palpation, and carries the potential of spreading along the fascial planes to the periorbital, mediastinal, pericardial, and/or thoracic spaces.^1^ Despite the fact that this condition may lead to life‐threatening complications,^4^ the majority of patients with this complication resolve spontaneously after 5‐10 days. Here, a case of sublingual emphysema subsequent to bone reduction with a high‐speed surgical handpiece is reported.

## 2 CASE REPORT

A 67‐year‐old Hispanic female presented for extraction of teeth numbered 32, 33, 42, and 43, alveoloplasty and placement of two implants for an overdenture. Her blood pressure was 135/78 mm Hg. The patient was a nonsmoker. She had a history of hypertension, rheumatoid arthritis, acid reflux, and asthma. Her medications included tramadol 50 mg, senna 8.6 mg, ranitidine 150 mg, prednisone 10 mg, pantoprazole 40 mg, ondansetron 4 mg, montelukast 10 mg, meloxicam 15 mg, gabapentin 300 mg, furosemide 20 mg, ferrous sulfate 325 mg, digoxin 250 μg, ipratropium‐albuterol 0.5‐3 mg, and fluticasone‐salmeterol 500 μg.

**Figure 1:**
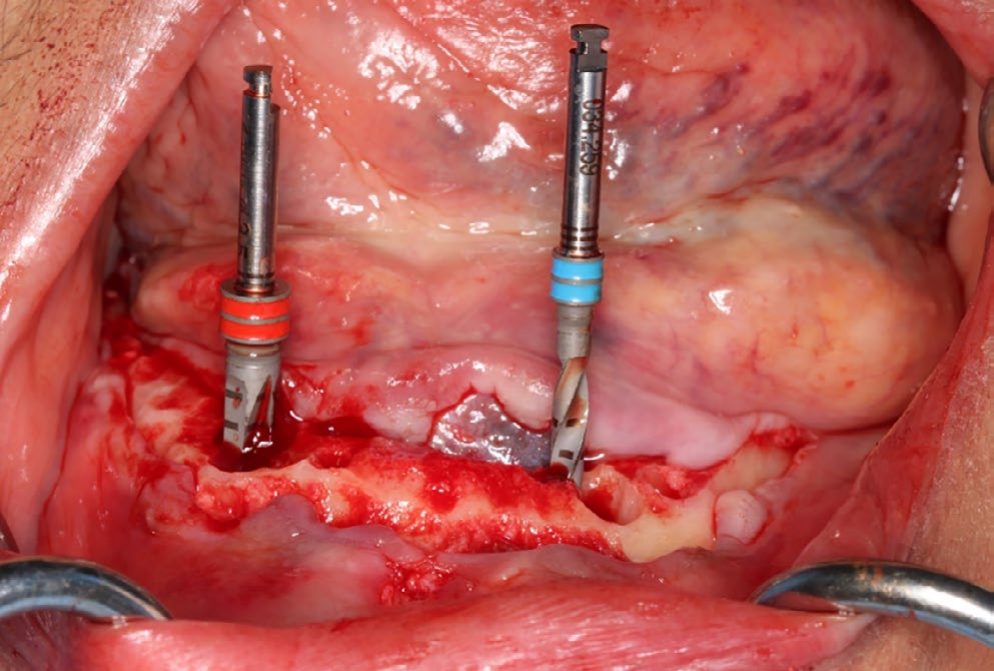
During the drilling process, slight swelling was noted in the floor of the mouth on the left side

Under local anesthesia, a full thickness flap was raised on the buccal and lingual aspect, extending from the distal of 33 to the distal of 43. The teeth were extracted uneventfully, and bone reduction to provide adequate restorative space for the overdenture was performed with a surgical air‐driven high‐speed surgical hand piece. During the drilling process, slight swelling was noted in the floor of the mouth on the left side (Figure 1). This was attributed to pressure and trauma from flap retraction as the patient was asymptomatic during the procedure. Implant drilling was continued uneventfully according to protocol, and two Straumann BLT implants 4.1 mm × 12 mm were placed in the region of 33 and 43. No dehiscences or fenestrations were created during osteotomy preparation, and adequate bone volume was present surrounding the implants.

Upon completion of the procedure, blood pressure was 132/86 mm Hg. A prescription for amoxicillin 500 mg, three times daily for seven days, and chlorhexidine mouthwash twice daily was given. Since she was already taking tramadol and meloxicam for arthritis‐related pain, no additional analgesics were prescribed. At the end of the procedure, there was no increase in the swelling previously noted on the left floor of the mouth and the patient was asymptomatic; therefore, she was discharged with postoperative instructions.

**Figure 2:**
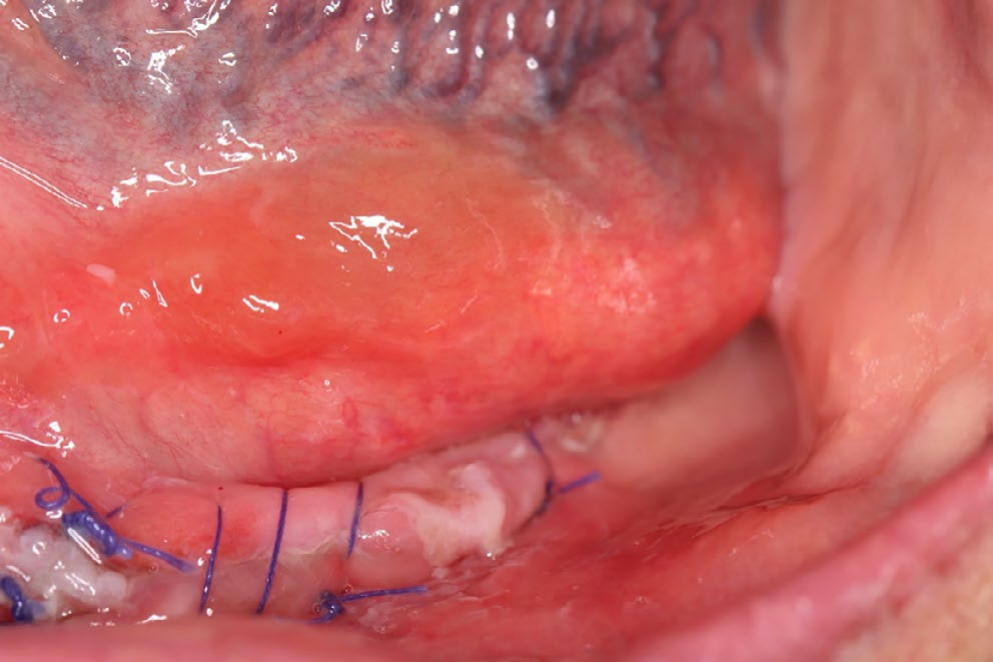
Swelling on the floor of the mouth covering the alveolar ridge on the left side

The patient reported the next day for denture insertion with the chief complaint of severe pain and swelling beneath her tongue on the left side. Her temperature was 98.5°F, and her blood pressure was 145/80 mm Hg. She said the pain began during the afternoon of the procedure immediately “after the numbness went away.” She also reported, “It feels like my tongue is sitting on something.” After physical examination, a marked edema of the left floor of the mouth was noted. The edema was present at the lateral incisor/canine region and extended from the original intra‐surgical location into the floor of the mouth. The edema was pronounced enough that it caused the lingual mucosa to cover the left alveolar ridge (Figure 2).

**Figure 3:**
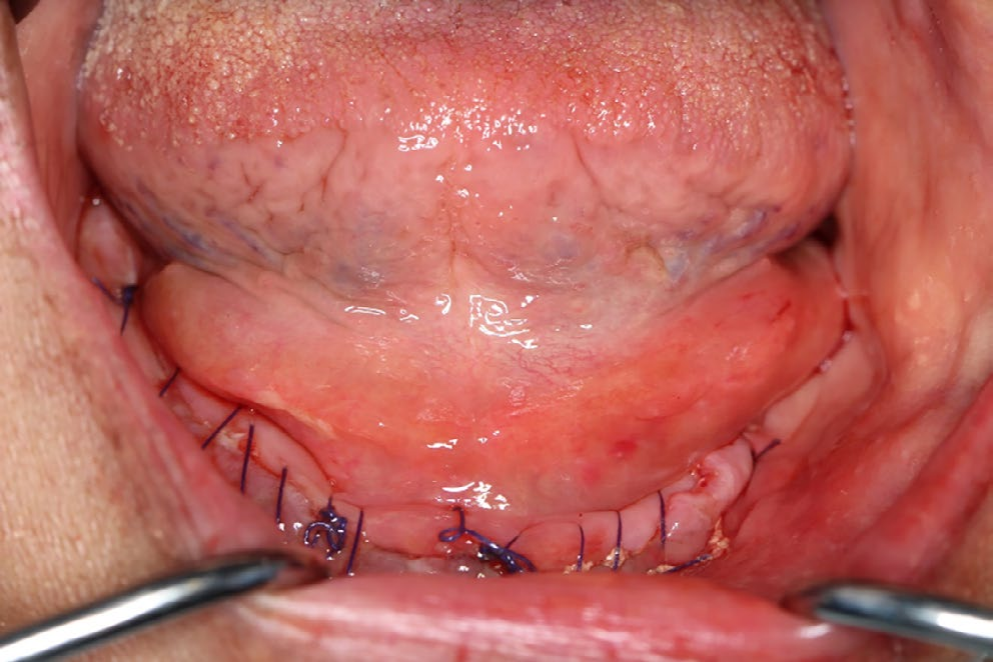
Reduction of swelling after incision and application of pressure

A pathological consult was done, and a differential diagnosis of emphysema or mucocele was made. Upon palpation, crepitus was felt. Under local anesthesia, a 5 mm superficial incision in the central portion of edematous area was made. There was no discharge of mucous from the incision, but the swelling reduced after light pressure was applied and air bubbles were seen in the heme surrounding the incision line (Figure 3). As the patient was taking antibiotics and using antiseptic mouthwash, she was dismissed and instructed to report the next day.

**Figure 4:**
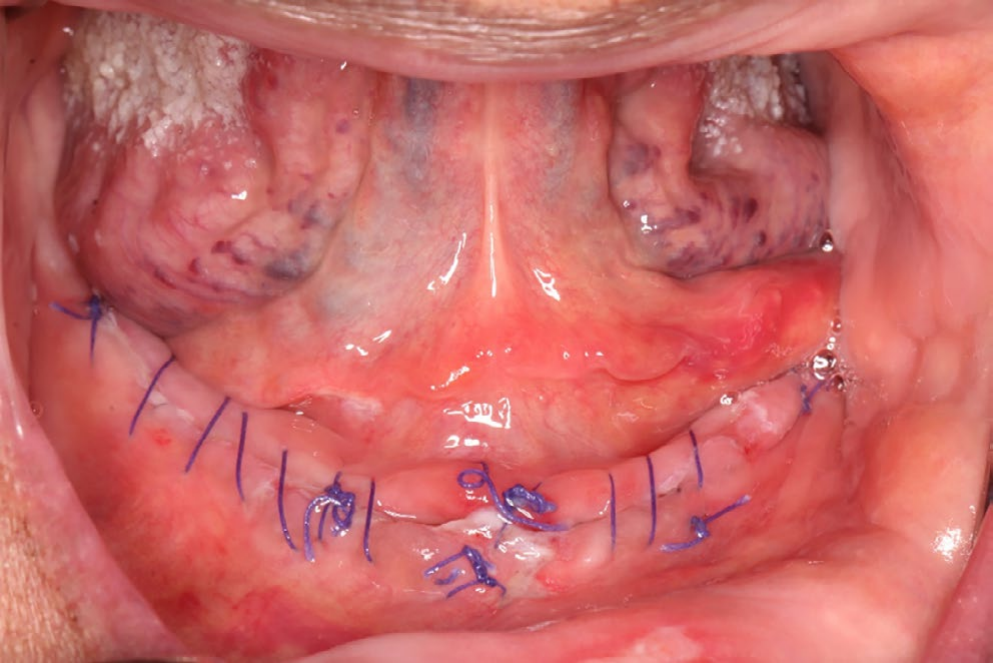
Further reduction of swelling during postoperative visit at 24 h

The next day the swelling was further reduced, the incision line appeared to be healing uneventfully by primary intention, and the patient denied any discomfort or pain (Figure 4).

**Figure 5:**
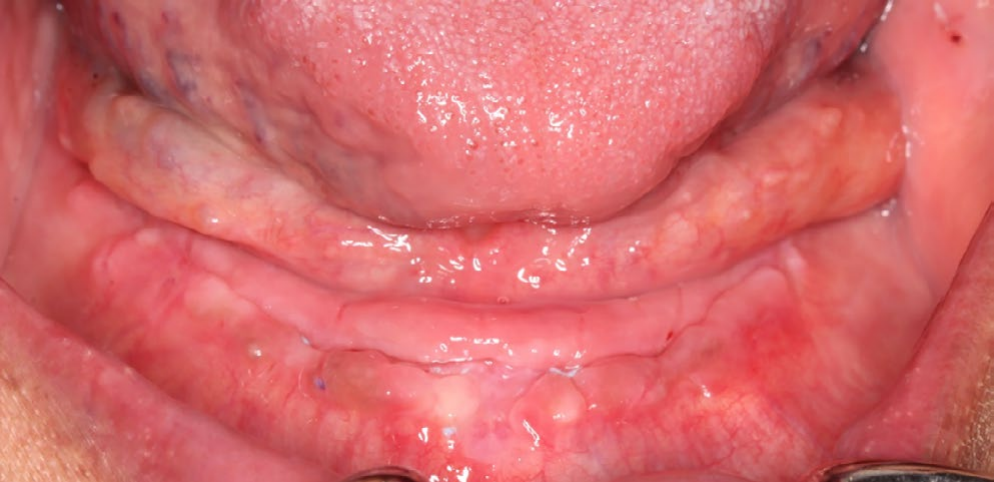
Complete resolution of the lesion at two weeks postoperative

She was advised to complete the course of antibiotics and rinse with chlorhexidine. At the 2‐week follow‐up, uneventful wound closure was observed clinically and sites of both emphysema and the original surgical area appeared within normal limits (Figure 5).

## 3 DISCUSSION

This case report described the manifestation of a sublingual emphysema after tooth extraction, alveoloplasty with a surgical air‐driven high‐speed handpiece, and implant placement. Emphysema related to dental treatment is caused by the entry of air or gas into the soft tissues through a break in mucosal integrity.^1^ In previous case reports, emphysema has been associated with the use of hydrogen peroxide,^4^ air turbine handpieces (Horowitz),^3^ air‐water syringes,^5^ dental extractions where an increased amount of intraoral pressure was used,^6^ and other intraoral injury.^7^ In the present case, it is believed that air entered the sublingual space during alveoloplasty, when the surgical handpiece head was inadvertently covered by the flap.

The most common clinical signs and symptoms of emphysema include a soft normal‐colored swelling, pain, and crepitus on palpation.^1^ Initial signs occur during or within an hour postsurgically in over 90% of the reported cases.^8^ Emphysema is usually restricted to moderate local swelling only; therefore, many cases are unrecognized or misdiagnosed. However, air that is trapped in the soft tissues may rapidly travel through fascial spaces and cause pneumopericardium, tension pneumothorax, air emboli, and airway compromise.^1^ Soft tissue infection is also possible, when bacteria are inserted in the tissues along with air.^9^

The differential diagnosis of submucous emphysema includes hematoma, soft tissue infection, and mucocele. When extensive subcutaneous involvement is present, angioedema and allergic reaction should also be considered. Swelling due to hematoma is characterized by rapidly progressing edema during surgery that appears red or blue due to the extravasation of heme. Soft tissue infection is accompanied by systemic symptoms such as fever, lymphadenopathy, and/or a change in hematological markers.^8^ Finally, a mucocele appears clinically similar to a localized submucous emphysema; however, the mucous‐filled swelling is easily displaced following slight pressure and no sound is evident. In cases of emphysema, the presence of crepitus on palpation is the determining factor that lead to the final diagnosis, as it is absent in all other conditions. The patient presented with localized superficial swelling in the sublingual space, pain, and crepitus. Therefore, conclusion of a final diagnosis was easily made by excluding other conditions.

Early diagnosis and management are crucial for the treatment of an emphysema. Although the majority of cases resolve spontaneously within 5‐10 days, some can lead to potentially life‐threatening complications.^1^ In such cases, antibiotic coverage, close clinical and, if needed, radiographic monitoring are necessary. A narrow‐spectrum antibiotic targeting normal oral flora, such as penicillin, is recommended to prevent a soft tissue infection that could arise if the trapped air carried bacteria into the tissues.^10^ Analgesics are prescribed as needed for the patient's comfort. In the present case, surgical intervention was selected to release the trapped air because the patient was already taking an antibiotic, amoxicillin, and an analgesic (nonsteroidal anti‐inflammatory drug) since the end of the surgical procedure. This unique decision to incise and drain the site was made due to the patient's reported tongue elevation and the risk for airway obstruction; as well as, ease of access of this localized and superficial emphysema. A linear incision was done in the floor of the mouth at the center of the swollen area and air was released. The emphysema and its surgical treatment did not complicate the outcome of the original procedure, as healing was within normal limits. However, in more extensive cases, surgical intervention is not recommended because of the risk for air movement through the fascial spaces and additional compromise to the patient. Lastly, prevention of emphysema seems the most prudent way of addressing this clinical complication. Careful, adequate flap elevation and retraction, and taking care to avoid contact between the flap and head of the handpiece are techniques that can be used to avoid emphysema. Even though surgical handpieces are the gold standard for alveoplasty due to the decreased amount of air released compared to conventional air‐driven handpieces, different instruments should be considered. Use of electrical handpieces, electrical bone saws, piezosurgical instruments, and rongeurs could be an alternative when performing alveoplasty, especially when working in close proximity to the floor of the mouth.

## 4 CONCLUSION

Emphysema in dentistry usually occurs with the use of air‐driven high‐speed handpieces or air syringes during dental, oral surgery, operative, endodontic or periodontal treatment. During surgical procedures, gentle maneuvers and adequate flap elevation and retraction in order to avoid handpiece head coverage by the flap are necessary for prevention of emphysema. Use of electrical handpieces, electrical bone saws, piezosurgical instruments, and rongeurs should be considered as alternative options to the use of air‐driven high‐speed instruments. It is important to have a differential diagnosis of this complication with other conditions that can produce swelling, like hematoma, soft tissue infection, mucocele, allergic reaction, or angioedema.^11^ In order to reach a correct diagnosis, a detailed history of the case is important, as well as a meticulous palpation of the involved tissue. Crepitus is the most important sign that differentiates emphysema from other pathological conditions.^12^

## CONFLICT OF INTEREST

The authors have no conflict of interest.

## AUTHOR CONTRIBUTIONS

AD: performed surgery and provided surgical details. KK: served as a case manager and restorative dentist. VK: performed literature review. SYR: performed literature review and edited the manuscript.
